# Effects and molecular mechanisms of *Achyranthes bidentata* Blume and *Cyathula officinalis* K.C. Kuan in the treatment of rheumatoid arthritis

**DOI:** 10.3389/fphar.2025.1619776

**Published:** 2025-07-24

**Authors:** Liu-Bo Zhang, Yu Yan, Wen-Wen Lian, Wei Zhou, Cong-Yuan Xia, Jun He, Yuan Xu

**Affiliations:** ^1^ National Center for Integrative Medicine, Department of TCM Rheumatism, Department of Pharmacy, China-Japan Friendship Hospital, Beijing, China; ^2^ Department of Rheumatology, The First Hospital of Shanxi Medical University, Taiyuan, China

**Keywords:** rheumatoid arthritis, *Achyranthes bidentata* Blume, *Cyathula officinalis* K.C.Kuan, pharmacology, pharmacokinetics

## Abstract

*Achyranthes bidentata* Blume (ABB; Chinese name: Huai Niuxi) and *Cyathula officinalis* K.C.Kuan (COK; Chinese name: Chuan Niuxi), two botanical drugs collectively termed “Niuxi” in traditional Chinese medicine (TCM), are widely used for rheumatoid arthritis (RA) management. This review comprehensively summarized the pharmacological mechanisms and therapeutic potential of the metabolites of ABB and COK on RA, while addressing limitations of current evidence. Of the 314 and 185 metabolites contained in ABB and COK, respectively, 22 metabolites (including Chikusetsusaponin V and chikusetsusaponin Ⅳa), showed multiple anti-RA activities. The mechanisms underlying the effects of ABB and COK with respect to the occurrence and development of RA (including inflammatory processes, immunoregulation, fibroblast-like synoviocytes, angiogenesis, oxidative stress, cartilage degradation, and bone destruction) were evaluated (Graphical Abstract). Numerous signaling pathways, such as the nuclear factor kappa-B (NF-κB), mitogen-activated protein kinase (MAPK), and phosphoinositide 3-kinase/protein kinase B (PI3K/AKT), are involved in RA. The metabolites contained in ABB and COK have significant medicinal value and potential in the treatment of RA, while in-depth mechanism studies and clinical research are warranted to support the clinical application of these metabolites.

## Graphical Abstract

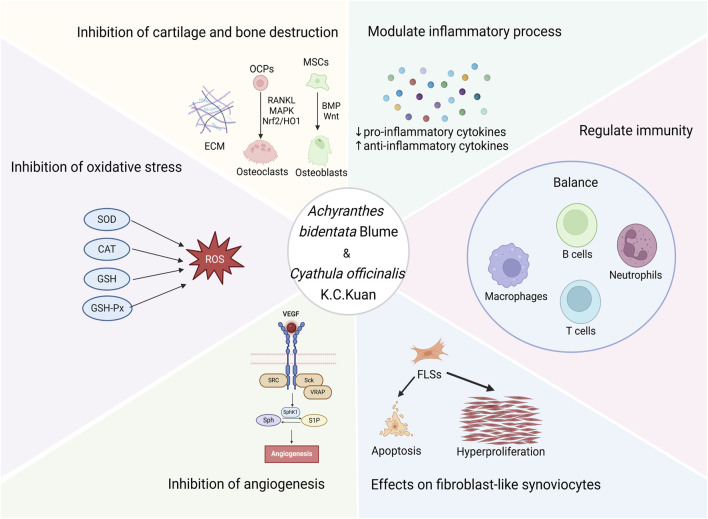

## 1 Introduction

Rheumatoid arthritis (RA) is an inflammatory autoimmune disease characterized by synovial inflammation, affecting approximately 1% of the population worldwide (van der Woude and van der Helm-van Mil, 2018). In the absence of treatment, RA can result in severe joint loss, deformity, and a high mortality rate (Babiker-Mohamed et al., 2024). Current treatments include non-steroidal anti-inflammatory drugs, glucocorticoids, disease-modifying antirheumatic drugs, and other immuno-modifiers (Baig et al., 2024). Although these drugs are effective against RA, approximately 40% of patients do not achieve clinical remission (Gao et al., 2024). Thus, there is an urgent need to development more efficacious anti-RA agents. Traditional Chinese medicine (TCM) has recently gained considerable attention due to the low cost, good safety profile, and multi-targeting nature of its medications (Wang et al., 2024a). TCM may be an important complementary medical strategy for RA.

“Niuxi” is one of the most commonly used medications in the TCM treatment of RA, with effects such as tonifying the liver and kidneys, strengthening muscles and bones, and relieving swelling and pain (Gao et al., 2013; Liu et al., 2013). Over 100 prescriptions for treating RA contain “Niuxi”, including Biqi capsule (Lv et al., 2023), Sanhan Chushi decoction (Ma et al., 2024), Qushi Zhitong Pills (Zhang et al., 2023). A meta-analysis of 15 studies involving 1,636 patients with RA showed that, compared with Western medicine, treatment with “Niuxi” and other TCMs, markedly increased the total effective rate and reduced the number of swollen joints, number of painful joints, rheumatoid factor (RF) levels, erythrocyte sedimentation rate (ESR), and C-reactive protein (CRP) levels (Lv et al., 2023). In a randomized controlled trial, compared to methotrexate and leflunomide, treatment with “Niuxi” and other TCMs combined with methotrexate and leflunomide in patients with RA obviously decreased CRP levels, ESR, tumor necrosis factor-α (TNF-α), interleukin (IL)-6, IL-17, RF, matrix metalloproteinase-3 (MMP-3) and transforming growth factor-β (TGF-β) (Chen et al., 2024). The National Medical Products Administration also approved numerous Chinese Patent Medicines containing “Niuxi” (e.g., Biqi capsules, Yuxuebi capsules, and Jishengshenqi pills) for the treatment of RA. A Bayesian network meta-analysis of 16 randomized controlled trials involving 10,213 patients with RA showed that Biqi capsules, Yuxuebi capsules, and glucosides of Tripterygium Wilfordii tablets were recommended for treating RA according to their clinical efficacy (Zhang et al., 2020a). *Achyranthes bidentata* Blume (ABB; Chinese name: Huai Niuxi) and *Cyathula officinalis* K.C.Kuan (COK; Chinese name: Chuan Niuxi) are both used as ‘Niuxi’. ABB and COK have been widely used to treat patients with RA in combination with other botanical drug, some of which are listed in Table 1.

**TABLE 1:** Classical prescriptionss of ABB or COK for treating RA in China.

Prescription name	Study design	Patients, treatment and duration	Outcome measures	Results	References
Biqi Capsules (痹祺胶囊)^a^	Randomized controlled trial	70 (1:1, the control group: Leflunomide and Methotrexate group; the observation group: Biqi Capsules and Methotrexate group) 24 weeks	20% improvement in ACR criteria (ACR20)	81.2% patients in Biqi group achieved ACR20 at 24 weeks. There were no significant differences in primary or secondary outcomes between the two groups	Wang et al. (2020)
	Randomized controlled trial	200 (1:1, the control group: Etoricoxib Tablets; the observation group: Etoricoxib Tablets and Biqi Capsules) 12 weeks	Total clinical effective (joint pain, morning stiffness, and swelling); CRP, RF, anti-streptolysin O (ASO), ESR, IgA, IgG, IgM, complement C3 and C4	The total clinical effective rate of observation group (83.00%) was significantly higher than control group (69.00%)	Liu et al. (2021b)
Yuxuebi Tablets (瘀血痹片)^b^	Randomized controlled trial	122 (1:1, the control group: Methotrexate Tablets; the observation group: Methotrexate Tablets and Yuxuebi Tablets) 12 weeks	LKSS, VAS, activies of daily living (ADL), IL-1, TNF-α, intercellular adhesion molecule-1 (ICAM-1)	The total effective rate of the observation group (93.44%) was higher than that of the control group (78.69%)	Fang et al. (2019)
Angelicae pubescentis and Loranthi Pills (独活寄生丸)^c^	Randomized controlled trial	120 (1:1:1, the control group: Celecoxib Capsules; the observation group 1: Angelicae pubescentis and Loranthi Pills; te observation group 2: Celecoxib Capsules and Angelicae pubescentis and Loranthi Pills) 14 days	IL-6 and TNF-α	It was significantly lower in the observation group 2 than that in the control group and observation group 1, and there was no statistical significance on the difference between observation group 1 and control group	Ding et al. (2021)
Tongbi Tablets (通痹片)^d^	Randomized controlled trial	132 (1:1, the control group: Celecoxib Capsules; the observation group: Celecoxib Capsules and Tongbi Tablets) 12 weeks	VAS, WOMAC and inflammatory cytokines (IL-1, TNF-α, ICAM-1)	The total effective rate of the observation group (95.45%) was higher than that of the control group (78.79%)	Su et al. (2020)
Tongbi Capsules (通痹胶囊)^d^	Randomized controlled trial	90 (1:1, the control group: Methotrexate Tablets and Leflunomide; the observation group: Methotrexate Tablets, Leflunomide and Tongbi Capsules) 4 months	Microcirculation index [resistance index (RI), end diastolic velocity (EDV), peak systolic velocity (PSV)], MMP-1, MMP-3, RF, CRP, ESR, ASO	The total effective rate of the observation group (95.56%) was higher than that of the control group (82.22%)	Li et al. (2020)
Fengshi Maqian Tablets (风湿马钱片)^e^	Retrospective study	84 (the control group: Methotrexate Tablets; the observation group: Methotrexate Tablets and Fengshi Maqian Tablets) 12 weeks	Joint pain, morning stiffness, and swelling; the levels of CRP, IL-17, TNF-α, COX-2 and Tumor necrosis factor-like ligand 1A (TL1A)	The total effective rate of the observation group (90.48%) was higher than that of the control group (71.43%)	Liu (2017)
Modified Simiao Pills (四妙丸加味)^f^	Retrospective study	92 (the control group: Leflunomide; the observation group: Leflunomide and Modified Simiao Pills) 1 month	DAS28, VAS, morning stiffness time, tenderness index and IL-1β, IL-6, TNF-α, ESR, PV (plasma viscosity), PCV (packed cell volume), IgG, IgA, IgE, CRP, RF	The total effective rate of the observation group (91.30%) was higher than that of the control group (78.26%)	Zhang et al. (2020b)
San Miao San (三妙散)^g^	Double-blind, randomized, placebo-controlled study	the TCM group (combination of Ganoderma lucidum (4 gm) and San Miao San (2.4 gm) daily) 32 cases and the placebo control group 33 cases 24 weeks	ACR20, plasma levels (percentage, absolute counts, and CD4+/CD8+/natural killer/B lymphocytes ratio, IL-18, INF-γ, MCP-1, RANTES)	15% in the TCM group compared with 9.1% in the placebo group achieved ACR20 (*P* > 0.05). Pain score and patient’s global score improved significantly only in the TCM group. The plasma levels were unchanged	Li et al. (2007a)

^a^ *Strychnos nux-vomica* L. [Loganiaceae, Strychni semen] 24.84 g, *Pheretima aspergillum* (E. Perrier) [Megascolecidae, Pheretima] 2.48 g, *Codonopsis pilosula* (Franch.) Nannf. [Campanulaceae, Codonopsis radix] 37.27 g, *Poria cocos* (Schw.) Wolf [Smilacaceae, Poria] 37.27 g, *Atractylodes macrocephala* Koidz. [Asteraceae, Atractylodis macrocephalae rhizoma] 37.27 g, *Glycyrrhiza glabra* L. [Fabaceae, Glycyrrhizae radix et rhizoma] 37.27 g, *Conioselinum anthriscoides ‘Chuanxiong’* [Apiaceae, Chuanxiong rhizoma] 49.69 g, *Salvia miltiorrhiza* Bunge [Lamiaceae, Salviae miltiorrhizae radix et rhizoma] 24.84 g, *Panax notoginseng* (Burkill) F.H.Chen [Araliaceae, Notoginseng radix et rhizoma] 24.84 g, and *Achyranthes bidentata* Blume [Amaranthaceae, Achyranthis bidentatae radix]24.84 g.

^b^ *Boswellia sacra* Flück. [Burseraceae, Olibanum], *Clematis chinensis* Osbeck [Ranunculaceae, Clematidis radix et rhizoma], *Carthamus tinctorius* L. [Asteraceae, Carthami flos], *Salvia miltiorrhiza* Bunge [Lamiaceae, Salviae miltiorrhizae radix et rhizoma], *Commiphora myrrha* (T.Nees) Engl. [Burseraceae, Myrrha], *Cyathula officinalis* K.C.Kuan [Amaranthaceae, Cyathulae radix], *Conioselinum anthriscoides ‘Chuanxiong’* [Apiaceae, Chuanxiong rhizoma], *Angelica sinensis* (Oliv.) Diels [Apiaceae, Angelicae sinensis radix], C*urcuma longa* L. [Zingiberaceae, Curcumae longae rhizoma], *Cyperus rotundus* L. [Cyperaceae, Cyperi rhizoma], and *Astragalus mongholicus* Bunge [Fabaceae, Astragali radix] (dosage information is not available).

^c^ *Angelica biserrata* (R.H.Shan and C.Q.Yuan) C.Q.Yuan and R.H.Shan [Apiaceae, Angelicae pubescentis radix]54 g, *Taxillus chinensis* (DC.) Danser [Loranthaceae] 54 g, *Gentiana macrophylla* Pall. [Gentianaceae, Gentianae macrophyllae radix] 54 g, *Saposhnikovia divaricata* (Turcz. ex Ledeb.) Schischk. [Apiaceae, Saposhnikoviae radix] 54 g, *Asarum heterotropoides* F.Schmidt [Aristolochiaceae, Asari radix et rhizoma] 54 g, *Angelica sinensis* (Oliv.) Diels [Apiaceae, Angelicae sinensis radix] (wine-processed) 80 g, *Paeonia lactiflora* Pall. [Paeoniaceae, Paeoniae radix alba] 36 g, *Conioselinum anthriscoides ‘Chuanxiong’* [Apiaceae, Chuanxiong rhizoma] 54 g, *Rehmannia glutinosa* (Gaertn.) DC. [Orobanchaceae, Rehmanniae radix] 36 g, *Eucommia ulmoides* Oliv. [Eucommiaceae, Eucommiae cortex] (stir-frying with salt) 54 g, *Achyranthes bidentata* Blume [Amaranthaceae, Achyranthis bidentatae radix] 54 g, *Codonopsis pilosula* (Franch.) Nannf. [Campanulaceae, Codonopsis radix] 54 g, *Poria cocos* (Schw.)Wolf. [Smilacaceae, Poria] 54 g, *Glycyrrhiza glabra* L. [Fabaceae, Glycyrrhizae radix et rhizoma] 36 g, and *Neolitsea cassia* (L.) Kosterm. [Lauraceae, Cinnamomi cortex] 54 g.

^d^ *Scolopendra subspinipes mutilans* L. Koch. [Scolopendridae, Scolopendra] 4.42 g, *Buthus martensii* Karsch [Buthidae, Scorpio] 4.42 g, *Pheretima aspergillum* (E. Perrier) [Megascolecidae, Pheretima] 4.42 g, *Bombyx mori* Linnaeus [Bombycidae, Bombyx batryticatus] 4.42 g, *Zoacys dhumnades* [Colubridae, Zaocys] 4.42 g, *Gastrodia elata* Blume [Orchidaceae, Gastrodiae rhizoma] 4.42 g, *Panax ginseng* C.A.Mey. [Araliaceae, Ginseng radix et rhizoma]1.48 g, *Astragalus mongholicus* Bunge [Fabaceae, Astragali radix]17.72 g, *Angelica sinensis* (Oliv.) Diels [Apiaceae, Angelicae sinensis radix] 26.56 g, *Hansenia weberbaueriana* (Fedde ex H.Wolff) Pimenov and Kljuykov [Apiaceae, Notopterygii rhizoma et radix] 4.42 g, *Angelica biserrata* (R.H.Shan and C.Q.Yuan) C.Q.Yuan and R.H.Shan [Apiaceae, Angelicae pubescentis radix]4.42 g, *Saposhnikovia divaricata* (Turcz. ex Ledeb.) Schischk. [Apiaceae, Saposhnikoviae radix] 4.42 g, *Ephedra sinica* Stapf [Ephedraceae, Ephedrae herba] 4.42 g, *Neolitsea cassia (L.) Kosterm.* [Lauraceae, Cinnamomi cortex] 4.42 g, *Aconitum carmichaelii* Debeaux [Ranunculaceae, Aconiti lateralis radix praeparaia] 4.42 g, *Coix lacryma-jobi* var. ma-yuen (Rom.Caill.) Stapf [Poaceae, Coicis semen]126.56 g, *Prunus persica* (L.) Batsch [Rosaceae, Persicae semen] 8.86 g, *Carthamus tinctorius* L. [Asteraceae, Carthami flos] 5.90 g, *Commiphora myrrha* (T.Nees) Engl. [Burseraceae, Myrrha] 4.42 g, *Manis* 4.42 g, *Corydalis yanhusuo* (Y.H.Chou and Chun C.Hsu) W.T.Wang ex Z.Y.Su and C.Y.Wu [Papaveraceae, Corydalis rhizoma] (vinegar-processed) 4.42 g, *Paeonia × suffruticosa* Andrews [Paeoniaceae, Moutan cortex] 4.42 g, *Gypsophila vaccaria* (L.) Sm. [Caryophyllaceae, Vaccariae semen] 4.42 g, *Piper kadsura* (Choisy) Ohwi [Piperaceae, Piperis kadsurae caulis] 8.86 g, *Cyperus rotundus* L. [Cyperaceae, Cyperi rhizoma] (wine-processed) 4.42 g, *Dolomiaea costus* (Falc.) Kasana and A.K.Pandey [Asteraceae, Aucklandiae radix] 4.42 g, *Citrus × aurantium* f. aurantium [Rutaceae, Citri reticulatae pericarpium] 4.42 g, *Wurfbainia villosa* (Lour.) Škorničk. and A.D.Poulsen [Zingiberaceae, Amomi fructus] 3.70 g, *Liquidambar formosana* Hance [Altingiaceae, Liquidambaris resina] 4.42 g, *Chaenomeles speciosa* (Sweet) Nakai [Rosaceae, Chaenomelis fructus] 4.42 g, *Achyranthes bidentata* Blume [Amaranthaceae, Achyranthis bidentatae radix] 4.42 g, *Dipsacus asper* Wall. ex DC. [Caprifoliaceae, Dipsaci radix] 4.42 g, *Lycopodium clavatum* L. [Lycopodiaceae, Lycopodii herba] 4.42 g, *Rheum palmatum* L. [Polygonaceae, Rhei radix et rhizoma] 4.42 g and *Cinnabaris* [Sulfides, Cinnabaris] 4.42 g.

^e^ *Strychnos nux-vomica* L. [Loganiaceae, Strychni semen], *Bombyx mori* Linnaeus [Bombycidae, Bombyx batryticatus], *Boswellia sacra* Flück. [Burseraceae, Olibanum], *Commiphora myrrha* (T.Nees) Engl. [Burseraceae, Myrrha], *Buthus martensii* Karsch [Buthidae, Scorpio], *Achyranthes bidentata* Blume [Amaranthaceae, Achyranthis bidentatae radix], *Atractylodes lancea* (Thunb.) DC. [Asteraceae, Atractylodis rhizoma], *Ephedra sinica* Stapf [Ephedraceae, Ephedrae herba], *Glycyrrhiza glabra* L. [Fabaceae, Glycyrrhizae radix et rhizoma] (dosage information is not available).

^f^ *Phellodendron chinense* C.K.Schneid. [Rutaceae, Phellodendri chinensis cortex] 10–15 g, *Atractylodes lancea* (Thunb.) DC. [Asteraceae, Atractylodis rhizoma] 10–15 g, *Achyranthes bidentata* Blume [Amaranthaceae, Achyranthis bidentatae radix]15–30 g, *Coix lacryma-jobi* var. ma-yuen (Rom.Caill.) Stapf [Poaceae, Coicis semen] 15–30 g. According to the specific situation of patients, *Chaenomeles speciosa* (Sweet) Nakai [Rosaceae, Chaenomelis fructus], *Gentiana macrophylla* Pall. [Gentianaceae, Gentianae macrophyllae radix], *Carthamus tinctorius* L. [Asteraceae, Carthami flos], *Angelica sinensis* (Oliv.) Diels [Apiaceae, Angelicae sinensis radix], *Conioselinum anthriscoides ‘Chuanxiong’* [Apiaceae, Chuanxiong rhizoma], *Boswellia sacra* Flück. [Burseraceae, Olibanum], *Piper kadsura* (Choisy) Ohwi [Piperaceae, Piperis kadsurae caulis], *Clematis chinensis* Osbeck [Ranunculaceae, *Clematis chinensis* Osbeck] were added.

^g^ *Atractylodes lancea* (Thunb.) DC. [Asteraceae, Atractylodis rhizoma], *Phellodendron amurense* Rupr. [Rutaceae, Phellodendri chinensis cortex], *Achyranthes bidentata* Blume [Amaranthaceae, Achyranthis bidentatae radix] (dosage information is not available).

COK, the root of *C. officinalis* Kuan, is traditionally thought to have anti-blood stasis (Cao et al., 2017), anti-inflammation (Yang et al., 2022), and hepatoprotective properties (Meng et al., 2019); in addition, it is used in the treatment of orthopedic diseases (Park et al., 2011). Huang et al. reported that COK impacts the hematologic, urogenital, and immune systems (Huang et al., 2019). The chemical metabolites contained in COK can be roughly divided into triterpenoid saponins, steroid ketones, polysaccharides, and others (Liang et al., 2022). Current pharmacologic studies of COK have primarily focused on cyasterone, achyranthan and polysaccharides (Han et al., 2015; Feng et al., 2019; Yang et al., 2022).

ABB, the dry root of *A. bidentata (A*.*bidentata)* and *Achyranthes aspera* Linn (*A. aspera*), prevents osteoporosis (Jiang et al., 2014b), exerts neurotrophic and neuroprotective effects (Wang et al., 2013), inhibits myocardial ischemic/reperfusion-induced injuries (Tie et al., 2013) and possesses antitumor properties (Zhong et al., 2020). *A. aspera* aqueous extract offers significant protection against arthritis and joint inflammation (Chinnasamy et al., 2019). Researchers have found that RA is related to excessive osteoclast resorption and defective osteoblast production (Yan et al., 2020). *A. bidentata* alcohol extract increases the proliferative capacity and stimulates the osteogenic differentiation of osteoblasts (Hua and Zhang, 2019). Moreover, it can inhibit osteoclast differentiation (Jiang et al., 2014b). ABB contains saponins, steroids, flavonoids, alkaloids, polysaccharides, and polypeptides. Of these metabolites, ecdysterone, polysaccharides, polypeptides, and saponins are the most characteristic metabolites of ABB (Jiang et al., 2014b).

Previous reviews have discussed the traditional uses, phytochemistry, and pharmacological properties of the genus Achyranthes (Li and Li, 1998; He et al., 2017). Further investigations included high-performance liquid chromatography analysis, processing, extraction conditions, and the biological activity of the saponin metabolites of *Achyranthes* root (Kiuchi, 2022). This review focuses on the pathogenesis of RA and the effects and molecular mechanisms of ABB and COK for the treatment of RA. A literature search was performed, focusing on research published up to May 2025. In detail, the metabolites contained in ABB and COK were examined using evidence available in the Web of Science, PubMed, China National Knowledge Infrastructure (CNKI), and Traditional Chinese Medicine Systems Pharmacology Database and Analysis Platform (TCMSP) (https://tcmspw.com/tcmsp.php) (Ru et al., 2014). Subsequently, the metabolites involved in RA were further filtered. The objective of this review was to improve our understanding of the anti-RA effects of ABB and COK. We first summarized the mechanisms underlying the effects of ABB and COK by focusing on the pathogenesis of the inflammatory process, immunoregulation, fibroblast-like synoviocytes (FLS), angiogenesis, oxidative stress, cartilage degradation, and bone destruction in RA. Data on the pharmacokinetic characteristics of the metabolites of ABB and COK were also summarized. This review attempts to comprehensively summarize the available information on the multiple beneficial effects of the metabolites of ABB and COK on RA, and provide scientific evidence for its traditional use.

## 2 Effects of ABB and COK on RA

### 2.1 Extract of ABB and COK

In an adjuvant-induced arthritis (AIA) rat model, treatment with a saponin-rich fraction of *A. aspera* (50 and 100 mg/kg) for 28 days markedly suppressed paw swelling, reduced the arthritic score, and improved the pain threshold (Kothavade et al., 2015). Zheng et al. isolated and identified ABB saponins in *A. aspera* root and explored their activity in rats with collagen-induced arthritis (CIA). ABB saponins (150 and 300 mg/kg) dramatically decreased the paw volume of arthritis rats. At the dose of 300 mg/kg, ABB saponins significantly suppressed soft tissue swelling and periarticular bone destruction and improved arthritis symptoms such as synovial joint space narrowing, synovial hyperplasia, fiber tissue hyperplasia, inflammatory cellular infiltration, cartilage erosion, and bony hyperplasia destruction (Zheng et al., 2016). In a rat AIA model, the total saponin content of ABB (30, 60, 120 mg/kg) significantly suppressed paw swelling and redness/inflammation severity in addition to improving synovial hyperplasia, and inflammatory cell infiltration (Fu et al., 2021).

*E*cdysteroid-enriched fraction of COK could reduce the arthritis score, paw swelling, and improve histopathological deterioration, and suppress the levels of inflammatory factors, matrix metalloproteinases, and proteins expressed in rat synovial tissue (Huang et al., 2025).

### 2.2 Metabolites of ABB and COK

A total of 314 metabolites contained in ABB were searched in the literature and TCMSP database, including saponins, steroids, flavonoids, alkaloids, fatty acid, and lignan (Li et al., 2007b; Tao et al., 2019a; Teng et al., 2022; Wang et al., 2022a; Wang et al., 2022b; Yan et al., 2024). Moreover, 185 metabolites contained in COK were searched, including saponins, steroids, flavonoids, alkaloids, fatty acid, and lignan (Yan et al., 2024). Of those, 22 metabolites, mainly derived from the roots of ABB and COK, were related to the treatment of RA, comprising 7 terpenoids, 6 flavonoids, 3 steroids, 2 alkaloids, and 4 others (Table 2) (Chen et al., 2024b). Duan et al. reported that arthritis scores were markedly decreased by higenamine in CIA mice (Duan et al., 2016). Chikusetsusaponin Ⅳa decreased the arthritis index, paw thickness and number of swollen joints in CIA mice (Guo et al., 2021).

**TABLE 2:** Effects and mechanism of major metabolites of ABB and COK in the treatment of RA.

Metabolites	Source	Model	Dose	Positive control	Biological activities	Mechanisms	References
20-hydroxyecdysone	ABB	LPS-induced RAW 264.7 cells	12.5 and 25 μM	Indomethacin (2.5 ng/mL)	↓PGE-2	unknow	Yoo et al. (2017)
β-Sitosterol	ABB, COK	BMDMs CIA mice	5, 25 and 50 μM 20 and 50 mg/kg	—	↓iNOS, IL-1β, CD86, MHCII, IgG1 ↑Arg-1, IL-10, CD163, CD206	unknow	Liu et al. (2019)
		VEGF-induced HUVECs	10 and 20 μM	—	↓VEGFR2, p-VEGFR2	VEGF pathway	Qian et al. (2021)
Azelaic acid	ABB	AIA rats	20, 40, and 80 mg/kg	Piroxicam (10 mg/kg)	↓TNF-α, IL-17, IL-1β, IL-6, COX-2, PGE-2, 5-LOX, anti-ccp, ESR, CRP, platelets, WBCs, MDA ↑IL-4, IL-10, hemoglobin, RBCs, SOD, CAT, GSH	↓NF-κB	Sial et al. (2024)
Caffeic acid	ABB, COK	RANKL and TNF-α induced RAW264.7	0.1, 1, 10 and 100 μg/mL	—	↓osteoclastogenesis	↓NFATc1	Tang et al. (2006)
		IL-1β induced FLS CIA rats	1, and 10 μM 30 and 50 mg/kg	—	↓TNF-α, IL-6, PGE2, MMP-1	↓NF-κB pathway	Wang et al. (2017)
Chikusetsusaponin IVa	ABB, COK	LPS induced THP-1 cells	50, 100 and 200 μg/mL	—	↓TNF-α, IL-1β, IL-6, COX-2, iNOS	↓MAPK, NF-κB pathway	Wang et al. (2015)
		LPS-induced RAW264.7 cells	6.25, 12.5 and 25 μM	—	↓TNF-α, IL-1β, iNOS, NO	↓miR-155, NF-κB ↑GSK-3β	Xin et al. (2020)
		CIA mice	50 and 100 mg/kg	Dexamethasone (0.2 mg/kg)	↓TNF-α, IL-1β, IL-6, IFN-γ	↓JAK1/2/STAT3	Guo et al. (2021)
		LPS-induced RAW264.7 cells	3.125, 6.25, 12.5 μg/mL	—	↓TNF-α, IL-1β, IL-6, IL-10, COX-2, iNOS, NO, PGE-2	↓MAPK pathway	Xu et al. (2021)
Chikusetsusaponin V	ABB, COK	LPS induced RAW264.7 cells	0.1, 1, 10, 40 μM	—	↓NO, iNOS, TNF-α, IL-1β, CD14, TLR4	↓MAPK (p-ERK, p-JNK) pathway, NF-κB pathway	Wang et al. (2014)
Cichoric Acid	ABB	CIA rats	8, 16, and 32 mg/kg	Tripterygium glycosides table (10 mg/kg)	↓TNF-α, IL-1β, PGE-2, COX-2	↓p-NF-κB	Jiang et al. (2014a)
Coptisine	ABB	FLS	5, 10, 20 μM	—	↓proliferation, migration, and invasion of FLS	↓PSAT1, MAPK pathway	Xu et al. (2024)
Higenamine	COK	CIA mice	10 mg/kg	—	↓TNF-α, IL-1β, MDA, caspase-3/9 ↑GSH	↑Nrf2/HO-1 pathway	Duan et al. (2016)
Hyperoside	ABB	LPS induced FLS CIA mice	10, 50, 100 μM 25, 50 mg/kg	—	↓TNF-α, IL-6, IL-1, MMP-9	↓NF-κB pathway	Jin et al. (2016)
Kaempferitrin	COK	MH7A cells CIA mice	5, 10 and 20 μM 10 and 20 mg/kg	—	↓TNF-α, IL-6, IL-1β, MMP-1, MMP-3	↓NF-κB, Akt/mTOR pathway	Wang and Zhao (2019)
Kaempferol	ABB	IL-1β induced FLS	100 µM	—	↓MMP-1, MMP-3, COX-2, PGE2	↓NF-κB, MAPK pathway	Yoon et al. (2013)
		bone marrow cells	100 µM	—	↓TRAP	↓c-Fos, NFATc1, p-ERK, p-JNK, p-p38	Lee et al. (2014)
		CIA rats Treg cells	100 mg/kg 50 μM	—	↑FOXP3, CTLA4, IL-10	↓FOXP3 phosphorylation	Lin et al. (2015)
		CIA mice FLS	2 mg/kg 2, 10 and 25 μM	—	↓TNF-α, IL-17, IL-21, TRAP	bFGF/FGFR3/RSK2 pathway	Lee et al. (2018)
		CIA mice TNF-α induced FLS	50, 100, 200 mg/kg 10, 20 and 40 μM	—	↓MMP-1, MMP-3, MMP-9, MMP-13	↓MAPK pathway	Pan et al. (2018)
		CIA mice	200 mg/kg	Leflunomide (5 mg/kg)	IL-6, TNF-α, IL-1β, IFN-γ, IgG	↓gut microbiota (Lachnospiraceae) ↑gut microbiota (Bacteroidales_S24-7_group, Prevotellaceae, Erysipelotrichaceae, Staphylococcaceae, Alcaligenaceae)	Aa et al. (2020)
Maslinic acid	ABB	Collagen antibody-induced arthritis mice	200 mg/kg	—	↓TNF-α, IL-1β	↓Toll-like receptor signaling, leukotrienes through the glucocorticoid receptor	Shimazu et al. (2019)
Momordin Ib	ABB	LPS-induced RAW264.7 cells	11.28 ± 0.55 μM	—	↓NO	Unknow	Huang et al. (2021)
Momordin Ic	ABB, COK	LPS-induced RAW264.7 cells	6.25, 12.5 and 25 μM	—	↓TNF-α, IL-6, PGE-2	Unknow	Yoo et al. (2017)
Nobiletin	ABB, COK	CIA mice IL-1β induced FLS	15, 30, and 60 mg/kg 16, 32, and 64 μM	—	↓ADAMTS-4, ADAMTS-5	Unknow	Imada et al. (2008)
		CIA rats	100 and 400 mg/kg	—	↓TNF-α, IL-6, IL-1β, MCP-1	↓p38/NF-κB pathway	Yang et al. (2017)
		IL-21 induced MH7A cells	25 and 50 μM	—	↓TNF-α, IL-6, HMGB1, MMP-3, MMP-13, 4-HNE	↓JAK1/STAT3 pathway	Liu et al. (2020)
		MG-63 cells	10 and 20 μg/mL	—	↑BMP-2, COL-I, ALP, OCN, RUNX2, COL1A1	↑BMP-2/RUNX2 pathway	Pang et al. (2021)
Oleanolic acid	ABB, COK	RANKL-induced RAW264.7 cells	2.5, 5 and 10 μM	Estradiol (1 μM)	↓TRAP, MMP-9, cathepsin K	↓RANK ↑ERα/miR-503 pathway	Xie et al. (2019)
p-Coumaric acid	ABB, COK	AIA rats	100 mg/kg	Celecoxib (5 mg/kg)	↓TNF-α, IL-1β, IL-6, IL-17, MCP-1, RANKL, TRAP, iNOS, COX-2 ↑OPG	↓NF-κB, MAPK (JNK, p-JNK, ERK1/2) pathway	Neog et al. (2017)
Rhoifolin	ABB, COK	AIA rats	10 and 20 mg/kg	Indomethacin (10 mg/kg)	↓TNF-α, IL-1β, IL-6, MDA ↑GSH, GSH-Px, SOD	↓NF-κB pathway	Peng et al. (2020)
Stigmasterol	ABB	CIA rats	200 mg/kg	MTX (0.25 mg/kg)	↓TNF-α, IL-6, IL-1β, iNOS ↑IL-10	↓NF-κB p65, p38MAPK	Khan et al., 2020
		CIA rats	50, 100, and 200 mg/kg	Indomethacin (1.0 mg/kg)	↓IL-6, IL-1β, RANKL, ACP5, Cathepsin K	Unkown	Xie et al. (2023)
Ursolic acid	ABB	AIA mice	10, 20, 40, 80 and 160 mg/kg	Prednisolone (5 mg/kg)	↓TNF-α, IL-2, IFN-γ	↓Th1 cells ↑Th2 cells	Ahmad et al. (2006)
		CIA mice	150 mg/kg	—	↓TNF-α, IL-1β, IL-6, IL-21, CII-specific IgG	↓Th17 and B cell differentiation	Baek et al. (2014)
		FLS	10 μM	—	↓Mcl-1	↑SP1/Noxa	Kim et al. (2018)
Wogonin	ABB	LPS-induced HUVECs	1,10 and 100 μM	Thalidomide (30 μg/mL)	↓VEGF, VEGFR-2, IL-6	Unkown	Lin et al. (2006)
		CIA mice MH7A cells	40 mg/kg 10,20 and 40 μM	MTX (1 mg/kg) MTX (1 μM)	↓TNF-α, IL-1β, IL-6, MMP-3, MMP-9, α-SMA ↑IL-10, E-Cad	↓PI3K/AKT/NF-κB	Yang et al. (2024)

## 3 Mechanisms underlying the effects of ABB and COK on RA

### 3.1 Modulation of the inflammatory process

#### 3.1.1 Effects on pro-inflammatory cytokines

It is well established that increased levels of pro-inflammatory cytokines, such as IL-1β, TNF-α, and IL-6, as well as the imbalance of anti-inflammatory cytokines, such as TGF-β, IL-4, IL-10, and IL-13, lead to the development of RA (Cutolo et al., 2022).

TNF-α can induce the production of other inflammatory cytokines, attracting leukocytes and constructing a synovial inflammatory environment. The synovial inflammatory environment activates macrophages to produce additional pro-inflammatory cytokines, such as IL-6 and IL-1β, which increase synovial inflammation by recruiting and activating other innate immune cells (Kikyo, 2023). Moreover, pro-inflammatory cytokines initiate and maintain the production of further degradative enzymes and prostaglandins, promoting cartilage degradation, bone resorption, and osteoclastogenesis (Markovics et al., 2021). In addition, IL-17 activates multiple downstream signaling cascades that increase the levels of several inflammatory cytokines and chemokines (Adamopoulos et al., 2010). Furthermore, IL-17 enhances osteoblast-mediated bone resorption by promoting the activity of receptor activator of nuclear factor-kappa B ligand (RANKL), macrophage colony-stimulating factor (M-CSF), and prostaglandin E2 (PGE2), thereby increasing cathepsin K (CTSK) and MMP expression (Zhang F. et al., 2011; Ganesan and Rasool, 2017).

*In vivo* studies showed that 20-hydroxyecdysone (Sun et al., 2017), stigmasterol (Khan et al., 2020), azelaic acid (Sial et al., 2024), ursolic acid (Baek et al., 2014), higenamine (Duan et al., 2016), cichoric acid (Jiang et al., 2014a), p-Coumaric acid (Neog et al., 2017), maslinic acid (Shimazu et al., 2019), rhoifolin (Peng et al., 2020), nobiletin (Yang et al., 2017), kaempferitrin (Wang and Zhao, 2019), and kaempferol (Aa et al., 2020) reduced the production of IL-6, IL-1β, TNF-α, IL-21 and IL-17 (Table 2).

**FIGURE 1:**
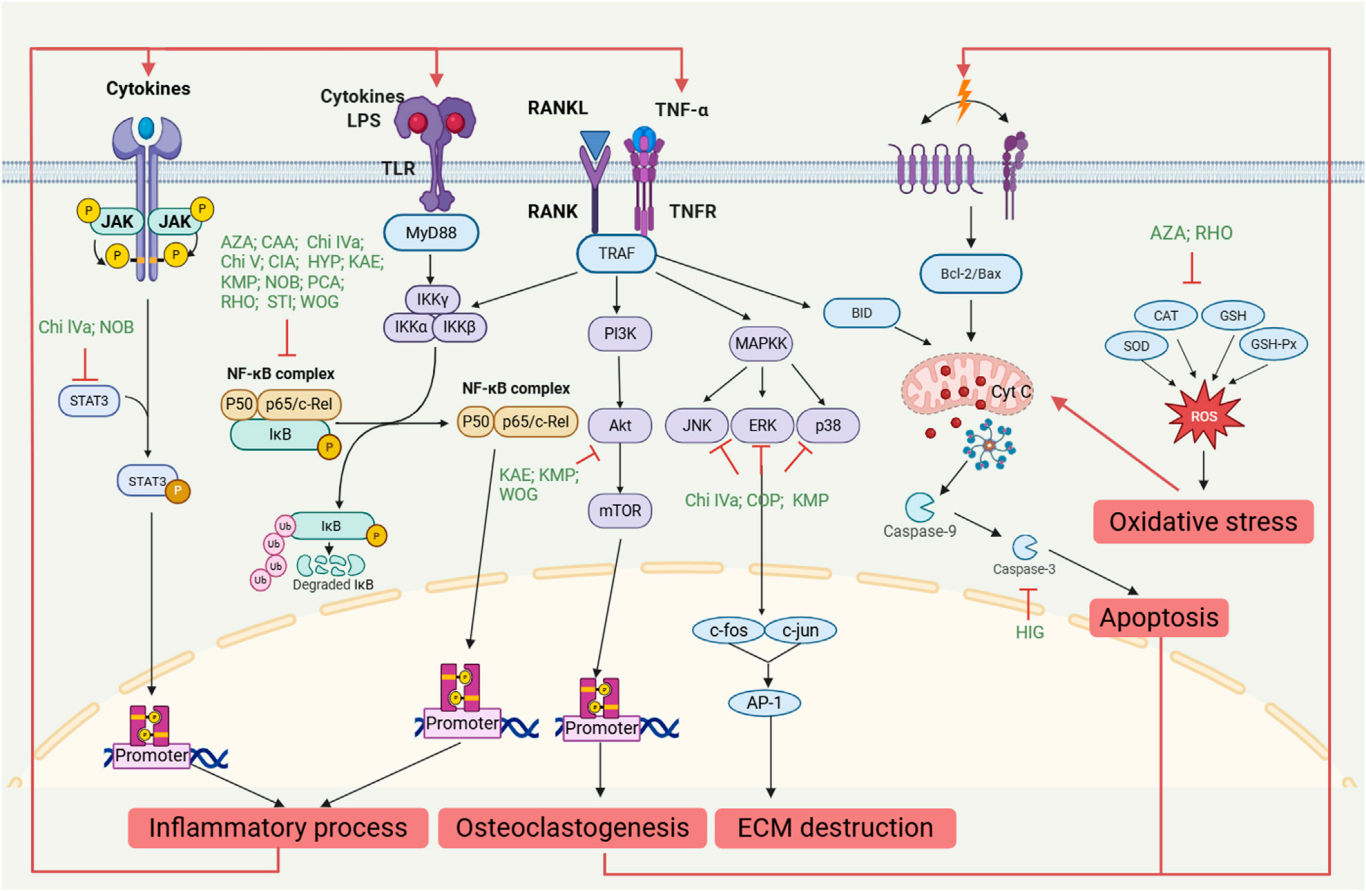
The molecular mechanisms ABB and COK on RA. AZA: Azelaic acid; CAA: Caffeic acid; Chi IVa: Chikusetsusaponin IVa; Chi V: Chikusetsusaponin V; CIA: Cichoric Acid; COP: Coptisine; HIG: Higenamine; HYP: Hyperoside; KAE: Kaempferitrin; KMP: Kaempferol; NOB: Nobiletin; PCA: p-Coumaric acid; RHO: Rhoifolin; STI: Stigmasterol; WOG: Wogonin.

Mechanistically, the primary signaling pathways involved in RA include Janus kinase (JAK), mitogen-activated protein kinase (MAPK), phosphoinositide 3-kinase (PI3K), and nuclear factor kappa-B (NF-κB) (Ding et al., 2023). Chikusetsusaponin IVa (Wang et al., 2015), chikusetsusaponin V (Wang et al., 2014), momordin Ic (Zheng et al., 2020), 20-hydroxyecdysone (Sun et al., 2017), stigmasterol (Khan et al., 2020), wogonin (Yang et al., 2024), caffeic acid (Wang et al., 2017), cichoric acid (Jiang et al., 2014a), p-Coumaric acid (Neog et al., 2017), rhoifolin (Peng et al., 2020), nobiletin (Yang et al., 2017), kaempferitrin (Wang and Zhao, 2019), hyperoside (Jin et al., 2016) suppressed the secretion of IL-1β, IL-6, IL-8, IL-17, and TNF-α via inhibiting NF-κB activation (Figure 1). Chikusetsusaponin IVa (Wang et al., 2015; Xu et al., 2021), chikusetsusaponin V (Xu et al., 2022), stigmasterol (Khan et al., 2020), caffeic acid (Wang et al., 2017), p-Coumaric acid (Neog et al., 2017), nobiletin (Yang et al., 2017) inhibited the production of pro-inflammatory cytokines by down-regulating the MAPK pathway (Figure 1). Moreover, wogonin and higenamine decreased the expression of TNF-α, IL-1β, and IL-6 via the PI3K/protein kinase B (PI3K/AKT) pathway (Duan et al., 2016; Yang et al., 2024). Chikusetsusaponin IVa could effectively and stably bind to IL-1β and interferon-gamma (IFN-γ) and inhibit the JAK/STAT signaling pathway (Guo et al., 2021) (Figure 1).

#### 3.1.2 Effects on anti-inflammatory cytokines

IL-10 can downregulate the expression of IL-1β, IL-6 and TNF-α, and reduce osteoclast formation by directly acting on osteoclast precursors (Kitaura et al., 2020). It was previously reported that β-sitosterol, azelaic acid, wogonin, and stigmasterol increased the expression of IL-10 (Liu et al., 2019; Khan et al., 2020; Sial et al., 2024; Yang et al., 2024). The mechanisms underlying these effects may be related to suppression of the MAPK, NF-κB, and PI3K/AKT pathways (Khan et al., 2020; Yang et al., 2024).

IL-4 exerted anti-inflammatory, anti-angiogenic, and bone-protective effects via suppressing pro-inflammatory cytokines, vascular endothelial growth factor (VEGF), osteoclast activation, and bone-resorbing cytokines (Iwaszko et al., 2021). In addition, production of IL-4 was increased by azelaic acid (Sial et al., 2024) (Table 2).

#### 3.1.3 Effects on nitric oxide (NO) and inducible nitric oxide synthase (iNOS)

NO is a significant inflammatory mediator overproduced in serum and synovial fluid of patients with RA, showing a significant correlation with disease activity (Farrell et al., 1992; Ali et al., 2014). Diverse cytokines including TNF-α and IL-6 increased the secretion of iNOS, subsequently promoting the production of NO by a broad spectrum of cells (e.g., macrophages, neutrophils, osteoclasts, osteoblasts, and FLS) into inflamed joints (Spiller et al., 2019; Huang et al., 2023). NO increased the production of inflammatory mediators via regulating the toll-like receptor 4/NF-κB (TLR4/NF-κB) pathway (Huang et al., 2023). Moreover, NO promoted the imbalance of T helper 17/regulatory T (Th17/Treg) cells, Th1/Th2 cells, and bone resorption/formation (Nagy et al., 2010; Spiller et al., 2019; Huang et al., 2023).

Chikusetsusaponin IVa (Wang et al., 2015; Xin et al., 2020), chikusetsusaponin V (Wang et al., 2014), momordin Ib (Huang et al., 2021), 20-hydroxyecdysone (Sun et al., 2017), β-Sitosterol (Liu et al., 2019), stigmasterol (Khan et al., 2020), ursolic acid (Baek et al., 2014), cichoric acid (Jiang L. et al., 2014), p-Coumaric acid (Neog et al., 2017), maslinic acid (Shimazu et al., 2019), nobiletin (Yang et al., 2017), and kaempferol (Aa et al., 2020) decreased the expression of iNOS and NO (Table 2).

#### 3.1.4 Effects on PGE2 and cyclooxygenase-2 (COX-2)

Apart from the above mechanisms, PGE2 formed by COX is a key inflammatory mediator in RA (Park et al., 2006). The production of PGE2 and COX-2 was increased by pro-inflammatory cytokines (e.g., TNF-α and IL-1β) and decreased by anti-inflammatory cytokines (e.g., IL-4, IL-10, and IL-13) (Martel-Pelletier et al., 2003). Evidence has shown that PGE2 is related to inflammation, angiogenesis and bone destruction by increasing the expression of IL-6 (Inoue et al., 2002), VEGF (Ben-Av et al., 1995), and MMP-1 (Kunisch et al., 2009).

Chikusetsusaponin IVa (Wang et al., 2015), chikusetsusaponin V (Kim et al., 2015), momordin Ic (Yoo et al., 2017), stigmasterol (Khan et al., 2020), azelaic acid (Sial et al., 2024), caffeic acid (Wang et al., 2017), cichoric acid (Jiang et al., 2014a), p-Coumaric acid (Neog et al., 2017), maslinic acid (Shimazu et al., 2019), nobiletin (Yang et al., 2017), kaempferol (Aa et al., 2020), and 20-Hydroxyecdysone (Yoo et al., 2017) suppressed the production of COX-2 and PGE2 (Table 2).

### 3.2 Regulation of immunity

#### 3.2.1 Effects on T cells

Through the induction of diverse cytokines, CD4^+^ T cells differentiate towards T cell subsets, including Th17, Treg, Th1, and Th2 (Gomez-Bris et al., 2023). Studies demonstrated that the imbalance of Th17/Treg and Th1/Th2 cells was closely related to inflammation and bone destruction in RA (Wang et al., 2024b; Yang and Zhu, 2024). Ursolic acid and kaempferol regulated the balance of Treg and Th17 cells by decreasing Th17 cells and increasing Treg cells (Baek et al., 2014; Lin et al., 2015; Lee et al., 2018). Ursolic acid decreased the cytokines of Th1 cells (Ahmad et al., 2006). Of note, ursolic acid increased the cytokines of Th2 cells (Ahmad et al., 2006) (Table 2).

#### 3.2.2 Effects on B cells

The functions of B cells include antigen presentation, cytokine secretion and autoantibody production, which are closely associated with the pathogenesis of RA (Wu et al., 2021a; Jang et al., 2022). *In vitro* studies showed that ursolic acid suppressed B cell activation and differentiation (Baek et al., 2014). Aa et al. reported that kaempferol decreased anti-collagen type II IgG levels in CIA mice (Aa et al., 2020). Liu et al. reported that β-sitosterol suppressed the levels of IgG and IgG1 antibodies, but did not affect those of IgG2c (Liu et al., 2019) (Table 2).

#### 3.2.3 Effects on macrophages

Macrophages play a crucial role in RA pathogenesis via polarization into M1 and M2 types (Yang et al., 2020). M1 macrophages produce pro-inflammatory cytokines (e.g., TNF-α, IL-6, IL-1β, IL-8, IL-12, and IL-23), while M2 macrophages produce anti-inflammatory cytokines (e.g., IL-10, IL-4, IL-13, and TGF-β) (Zheng et al., 2024). The proportions of M1 and M2 macrophages are imbalanced in the synovial fluid (Zhu et al., 2015), synovium (Ambarus et al., 2012) and peripheral blood (Tardito et al., 2019) in patients with RA. By modulating macrophage polarization, β-sitosterol inhibited the expression of iNOS, IL-1β, CD86, and major histocompatibility complex class II (MHCII), whereas it increased that of arginase-1 (ARG1), IL-10, CD163, and CD206 (Liu et al., 2019) (Table 2).

### 3.3 Effects on FLS

Proliferation of synovial tissue, an important feature of RA, is mainly attributed to hyperproliferation and decreased apoptosis of FLS (Tu et al., 2018). Coptisine (Xu et al., 2024), wogonin (Yang et al., 2024), kaempferitrin (Wang and Zhao, 2019), hyperoside (Jin et al., 2016), and kaempferol (Lee et al., 2018; Pan et al., 2018) suppressed the proliferation, migration, and invasion of FLS. Furthermore, caffeic acid (Wang et al., 2017), ursolic acid (Kim et al., 2018), and kaempferitrin (Wang and Zhao, 2019) induced apoptosis of FLS.

FLS have pro-inflammatory, angiogenic, and bone-destructive effects via overproduction of pro-inflammatory cytokines, chemokines, pro-angiogenic factors, collagenases, aggrecanases, cathepsins, and RANKL (Nygaard and Firestein, 2020; Wu et al., 2021b). It was reported that wogonin (Yang et al., 2024), caffeic acid (Wang et al., 2017), and hyperoside (Jin et al., 2016) suppressed the expression of TNF-α, IL-1β, IL-6, IL-8, IL-17 and IL-18 produced by FLS (Table 2).

### 3.4 Inhibition of angiogenesis

In RA, angiogenesis can be induced by inflammation, immune imbalance, and hypoxia, promoting synovial hyperplasia, pannus formation, and bone destruction (Wang et al., 2021). Angiogenesis is tightly regulated by VEGF and its receptors, which have been extensively studied (Khodadust et al., 2022). β-sitosterol and wogonin suppressed rheumatoid synovial angiogenesis via down-regulating vascular endothelial growth factor receptor 2 (VEGFR2) and phospho-VEGFR2 expression (Lin et al., 2006; Qian et al., 2021).

### 3.5 Inhibition of oxidative stress

In the arthritic joint, neutrophils can drive inflammation by releasing a variety of potentially harmful peptides, enzymes, and toxic oxygen metabolites (Cecchi et al., 2018). Myeloperoxidase, an abundant peroxidase in neutrophils, is found in the plasma and synovium of patients with RA (Fernandes et al., 2012; Odobasic et al., 2014). Moreover, increased oxidative stress decreases the levels of superoxide dismutase, catalase, glutathione, glutathione S-transferase, glutathione reductase, and glutathione peroxidase (Tang et al., 2022). The decrease in antioxidant defense is accompanied by a concurrent increase in the levels of ROS and NO (Wójcik et al., 2021). High levels of malondialdehyde indicate increased lipid peroxidation, which can activate signaling pathways involved in the inflammatory processes of RA (Tsikas and Mikuteit, 2022). Azelaic acid (Sial et al., 2024), 20-hydroxyecdysone (Sun et al., 2017), higenamine (Duan et al., 2016), and rhoifolin (Peng et al., 2020) can inhibit the decrease in superoxide dismutase, catalase, glutathione, glutathione peroxidase activity, and the increase in malondialdehyde content (Table 2).

### 3.6 Inhibition of cartilage and bone destruction

Under normal physiologic circumstances, bone remodeling is continuously conducted by osteoblasts and osteoclasts, which are responsible for bone resorption and bone formation, respectively. However, under arthritic conditions, the excessive activation of osteoclasts is associated with suppressed osteoblast development.

#### 3.6.1 Effects on osteoclasts

In RA, osteoclasts play a crucial role in bone resorption. Osteoclasts are formed by the fusion of osteoclast precursors, derived from monocyte/macrophage precursors or hematopoietic stem cells. TNF-α can regulate bone resorption by increasing RANKL and M-CSF expression in osteoblasts and stromal cells (Marahleh et al., 2019). The binding of M-CSF and colony-stimulating factor 1 receptor is essential for the survival, proliferation, differentiation and function of myeloid cells, including osteoclasts and monocytes/macrophages (Mun et al., 2020). Inhibition of osteoclastogenesis, osteoclast differentiation, and osteoclast function is a major target in the treatment of RA. Chikusetsusaponin IVa, momordin Ib and momordin IIa inhibited the formation of 1α,25(OH)_2_D_3_-induced osteoclast-like multinucleated cells in a co-culture system without irreversible toxicity (Li et al., 2005). Stigmasterol inhibited the expression of osteoclast-specific genes including cathepsin K, MMP-9 and tartrate-resistant acid phosphatase (TRAP) (Xie et al., 2023) (Table 2).

Upregulation of osteoclast activity in the development of RA is controlled by osteocytes through the osteoprotegerin/RANKL/RANK (OPG/RANKL/RANK) system (Zhang and Wen, 2021). RANKL binds to RANK on mononuclear osteoclast precursors. Subsequently, it recruits TRAF6, TGF-β activated kinase 1 binding protein 1 (TAB1), and TAB2 to sequentially activate TAK1 and promotes the activation of MAPKs, NF-κB and c-Fos. Finally, it induces the nuclear factor of activated T-cells cytoplasmic 1 (NFATc1), which is a crucial transcription factor of osteoclast differentiation (Maeda et al., 2022; Yang and Zhu, 2024). OPG, a decoy receptor of RANKL, blocks the RANKL-RANK interaction to inhibit osteoclast formation (Yao et al., 2021). Oleanolic acid significantly suppressed osteoclast differentiation and bone resorption via the estrogen receptor alpha/miR-503/RANK (ERα/miR-503/RANK) signaling pathway (Xie et al., 2019). Caffeic acid exerted an inhibitory effect on osteoclastogenesis by inhibiting the expression of NFATc1 (Tang et al., 2006). p-Coumaric acid inhibited osteoclastogenesis via up-regulating OPG expression and down-regulating RANKL, NFATc1 and c-Fos expression (Neog et al., 2017). Additionally, kaempferol attenuated osteoclast differentiation by suppressing the MAPK/c-fos/NFATc1 signaling pathway (Lee et al., 2014) (Table 2).

#### 3.6.2 Effects on osteoblasts

Osteoblasts are derived from mesenchymal stem cells in the bone marrow. These cells differentiate into osteoblasts, adipocytes or chondrocytes. Their differentiation into the osteogenic lineages is tightly controlled by molecular factors, such as bone morphogenic protein (BMP) and Wnt pathways (Datta et al., 2008; Zhang et al., 2011b). The canonical Wnt signalling pathway affects osteoblast proliferation and differentiation. MAPK is also thought to mediate the activation of several gene products related to bone formation, such as alkaline phosphatase (ALP) and transcription factors, including runt-related transcription factor 2 (RUNX2), osterix (OSX), and BMP2 (Hua and Zhang, 2019). Numerous reports over the last 2 decades showed that osteoblast differentiation also relies on miRNAs, and several miRNAs target the Wnt and BMP pathways, thereby modulating osteoblast differentiation (Ponzetti and Rucci, 2021). ALP activity is the gold standard for evaluating osteoblast differentiation. Chikusetsusaponin IVa was positively correlated with ALP activation, while chikusetsusaponin V and chikusetsusaponin IV were negatively correlated with ALP (Tao et al., 2019a). Nobiletin augmented the expression of type I collagen (COL-I), ALP, osteocalcin (OCN), and collagen type I alpha 1 chain (COL1A1) via promoting the BMP2/RUNX2 pathway (Pang et al., 2021) (Table 2).

#### 3.6.3 Effects on MMPs

RA is characterized by the loss of cartilage and bone matrix integrity (Komatsu and Takayanagi, 2022). Reversing the anabolic/catabolic imbalance of matrix remodeling is crucial to maintaining cartilage and bone integrity (Deng et al., 2022). Extracellular matrix (ECM) destruction occurs in the RA joint. The ECM primarily consists of type II collagen and proteoglycans. Matrix-degrading enzymes, such as MMPs and the A disintegrin and metalloproteinase with thrombospondin motif (ADAMTS) family degrade the ECM (Bian et al., 2023). ECM breakdown products (e.g., such as fibronectin fragments, tenascin C, and hyaluronic acid) can induce the production of pro-inflammatory cytokines, as well as MMP-3, MMP-9, and MMP-13, accelerating the breakdown of cartilage and bone. Inflammation can also promote the expression of MMPs, ADAMTS, and ROS, further promoting cartilage and bone destruction. Nobiletin (Imada et al., 2008; Liu et al., 2020), kaempferitrin (Wang and Zhao, 2019), and kaempferol (Yoon et al., 2013; Pan et al., 2018) significantly suppressed the expression of MMPs and ADAMTS family (Table 2).

## 4 Pharmacokinetic characteristics

Currently, there is limited number of pharmacokinetic studies of ABB extracts. Tao et al. studied the pharmacokinetics of chikusetsusaponin IV, chikusetsusaponin IVa and ginsenoside Ro in rats after oral administration of crude and salt-processed ABB. A double-peak phenomenon was observed for these three saponins. After oral gavage of crude and salt-processed ABB in rats, the highest values of the area under the curve up to the last quantifiable time-point (AUC_0-t_) and maximum concentration (C_max_) of the three saponins were observed in kidney tissue, followed by liver, spleen, heart, and lung tissues. The concentration of ginsenoside Ro and chikusetsusaponin IVa was higher in the raw extract than the salt-processed ABB. However, the C_max_ values of ginsenoside Ro and chikusetsusaponin IVa in rat plasma were lower in the raw group than the salt-processed group. This finding demonstrated that salt-processing resulted in enhanced bioavailability (Tao et al., 2018; Tao et al., 2019b).

Cyasterone, 25-epi-28-epi-cyasterone, precyasterone and capitasterone from COK phytoecdysteroids extract in normal and AIA rats were studied and the results demonstrated that the plasma concentrations of those metabolites were lower in AIA rats than in normal rats at almost all time points. The mechanism of the difference between normal rats and AIA rats with RA requires further research (Wang et al., 2023).

## 5 Safety assessment of ABB and COK

ABB is a botanical drug, commonly used to treat a variety of diseases. The oral toxicity and genotoxicity of single-dose (500, 1000, and 2000 mg/kg) and 4-week repeated-doses of water extract of ABB were evaluated. The single-dose oral toxicity study showed no mortality or treatment-related body weight changes, with the lethal dose in rats estimated to exceed 2000 mg/kg. The 4-week study on repeated oral doses showed that ARB did not lead to significant changes in body weight, organ weight, food intake, or hematological and serum biochemical parameters across all groups. Meanwhile, ABB did not induce genotoxicity in the chromosomal aberration test and micronucleus test (Hyun et al., 2021).

The mice displayed neurotoxic and gastrointestinal toxic effects after receiving a high dose of water extract of COK. The range of maximum tolerance doses for samples from different origins was between 34.9 and 56.8 g/kg (Huang et al., 2019). The recommended dosage for COK is 5–10 g in the National Medical Products Administration. Therefore, it is crucial to avoid high doses or prolonged use in clinical practice.

## 6 Discussion

RA is an inflammatory autoimmune disease. Currently drugs not only alleviate pain and swelling but also prevent damage to joint. Despite this, treating up to 20% of RA patients is challenging (de Hair et al., 2018), and these drugs can result in gastrointestinal bleeding and liver toxicity (Lin et al., 2020). TCM is gradually developing into an important complementary medical treatment strategy. ‘Niuxi’ is one of the most commonly utilized TCM treatment options for RA (Cai et al., 2024). Studies have shown that the metabolites contained in ABB and COK can significantly delay the progression of RA. However, some anti-RA effects of these metabolites have mostly remained at the *in vitro* level, further investigation is necessary to assess the therapeutic impact of metabolites contained in ABB and COK for the treatment of RA.

This review revealed that 22 metabolites contained in ABB and COK, including higenamine and chikusetsusaponin Ⅳa, exhibited anti-RA activity. These metabolites modulate the inflammatory process, regulate immunity, decrease the proliferation, migration, and invasion of FLS, induce apoptosis of FLS, inhibit angiogenesis and oxidative stress, and suppress the destruction of cartilage and bone. Many signaling pathways, such as NF-κB, MAPK, PI3K/AKT, and JAK/STAT, participate in the progression of RA (Table 2; Figure 1). NF-κB, MAPK, PI3K/AKT are important intracellular signaling pathways that play critical roles in the inflammation and bone destruction of RA (Liu et al., 2021a). TNF-α binds to TNFR and RANKL binds to RANK, recruiting TRAF and activating the PI3K/AKT, NF-κB and MAPK pathway (Ma et al., 2018; Shi and Sun, 2018). However, the research on its mechanism is still not in-depth. In the future, genomics, proteomics, and metabolomics and network pharmacology can be used to clarify the mechanism of ABB and COK for the treatment of RA, thereby enhancing its benefits to human health (Zhang et al., 2024; Lu, 2025). Meanwhile, the use of standardized animal models for RA, together with TCM symptom characteristics, is crucial for studying the molecular mechanisms of ABB and COK on RA (Zhou et al., 2023).

The present review indicates that the metabolites contained in ABB and COK may be a great complementary medicine option for RA. Although the mechanisms of metabolites contained in ABB and COK for treating RA were summarized via multiple targets and pathways, there is limited number of studies to explore combination therapy of metabolites from ABB or COK. It is necessary to explore combination therapy of metabolites from ABB or COK on RA in the future.

Chinese medicinal materials from animals, plants, and minerals must be treated properly before being used in decoctions. Such processes greatly influence the content and pharmacological activity of major metabolites (Han et al., 2019; Chen et al., 2020). ABB is generally prepared with yellow rice wine or salt water to enhance actions, such as enriching the kidneys, nourishing the liver, and strengthening the sinews and bones. Salt-processing leads to high levels of β-ecdysterone, 25-S inokosterone, 25-R inokosterone, chikusetsusaponin V, and chikusetsusaponin IVa, which were higher than that those detected in raw and wine-processed ABB (Yang et al., 2018). The levels of ginsenoside Ro and chikusetsusaponin IV were reduced, which could enhance the anti-osteoporotic effects of ABB (Tao et al., 2019a). The processing method has a significant influence on the content of each metabolite.

Previous studies have reported that the plasma concentrations of COK-derived phytoecdysteroids were lower in AIA rats than in normal rats (Wang et al., 2023). Various pathological mechanisms of RA may lead to changes in the absorption of metabolites, which requires further research.

In conclusion, ABB and COK metabolites represent have significant medicinal value and potential in the treatment of RA. Thus far, studies on the anti-RA activity of these metabolites have mostly remained at the *in vitro* level, and mechanism research is not in-depth enough, clinical research evidence is lacking. Therefore, in-depth mechanism studies and clinical research are warranted to support the clinical application of these metabolites.
